# Transcriptome Analyses Reveal IL6/Stat3 Signaling Involvement in Radial Glia Proliferation After Stab Wound Injury in the Adult Zebrafish Optic Tectum

**DOI:** 10.3389/fcell.2021.668408

**Published:** 2021-04-30

**Authors:** Yuki Shimizu, Mariko Kiyooka, Toshio Ohshima

**Affiliations:** ^1^Functional Biomolecular Research Group, Biomedical Research Institute, National Institute of Advanced Industrial Science and Technology, Osaka, Japan; ^2^DBT-AIST International Laboratory for Advanced Biomedicine, National Institute of Advanced Industrial Science and Technology, Osaka, Japan; ^3^Department of Life Science and Medical Bio-Science, Waseda University, Tokyo, Japan; ^4^Graduate School of Advanced Science and Engineering, Institute for Advanced Research of Biosystem Dynamics, Waseda Research Institute for Science and Engineering, Waseda University, Tokyo, Japan

**Keywords:** zebrafish, brain regeneration, optic tectum, radial glia, STAT3 signaling, transcriptome analysis

## Abstract

Adult zebrafish have many neurogenic niches and a high capacity for central nervous system regeneration compared to mammals, including humans and rodents. The majority of radial glia (RG) in the zebrafish optic tectum are quiescent under physiological conditions; however, stab wound injury induces their proliferation and differentiation into newborn neurons. Although previous studies have functionally analyzed the molecular mechanisms of RG proliferation and differentiation and have performed single-cell transcriptomic analyses around the peak of RG proliferation, the cellular response and changes in global gene expression during the early stages of tectum regeneration remain poorly understood. In this study, we performed histological analyses which revealed an increase in isolectin B4+ macrophages prior to the induction of RG proliferation. Moreover, transcriptome and pathway analyses based on differentially expressed genes identified various enriched pathways, including apoptosis, the innate immune system, cell proliferation, cytokine signaling, p53 signaling, and IL6/Jak-Stat signaling. In particular, we found that Stat3 inhibition suppressed RG proliferation after stab wound injury and that IL6 administration into cerebroventricular fluid activates RG proliferation without causing injury. Together, the findings of these transcriptomic and functional analyses reveal that IL6/Stat3 signaling is an initial trigger of RG activation during optic tectum regeneration.

## Introduction

Neural stem cells (NSCs) have been observed in the adult brains of all vertebrates and can continuously generate new neurons (Kizil et al., 2012; Balthazart and Ball, 2014; Berninger and Jessberger, 2016; Kempermann et al., 2018; Joven and Simon, 2018; McDonald and Vickaryous, 2018). The adult mammalian brain has two neurogenic regions, the subventricular zone (SVZ) and the subgranular zone in the hippocampus (Altman and Das, 1965; Eriksson et al., 1998), whereas the neurogenic regions of birds and reptiles are mainly located in the telencephalon (Balthazart and Ball, 2014; McDonald and Vickaryous, 2018). Conversely, the adult brains of teleosts contain many neurogenic niches (Zupanc and Horschke, 1995; Adolf et al., 2006; Grandel et al., 2006; Alunni et al., 2010); for instance, zebrafish have 16 different proliferative niches (Grandel et al., 2006) while medaka has 10 (Alunni et al., 2010). Moreover, mammals and birds display a limited central nervous system (CNS) regenerative capacity compared to amphibians and teleosts (Alunni and Bally-Cuif, 2016). In mammals, stroke activates neural progenitors in the SVZ which then migrate toward injured areas; however, functional recovery remains limited despite the presence of some newborn neurons (Yamashita et al., 2006; Kaneko et al., 2018). In contrast, urodele amphibians and teleosts have a high capacity for brain regeneration (Urata et al., 2018) and adult zebrafish have many neurogenic niches and display a high regenerative capacity in the CNS, including the brain, retina, and spinal cord (Becker et al., 1997; Adolf et al., 2006; Grandel et al., 2006; Raymond et al., 2006; März et al., 2011). Therefore, zebrafish are a well-studied model organism in CNS regeneration due to their high neurogenic potential, easy genetic manipulation, and the availability of genomic and transcriptomic data. In particular, various injury models in the brain, retina, and spinal cord of adult zebrafish have been developed to elucidate and analyze the molecular mechanisms that enable CNS regeneration (Ramachandran et al., 2010; Dias et al., 2012; Kishimoto et al., 2012).

Radial glia (RG), or ependymoglia (neurepithelial-like stem cells), have been reported to function as NSCs in the neurogenic niches of adult zebrafish (Than-Trong and Bally-Cuif, 2015). In the telencephalon, which is a well-studied region of the adult zebrafish brain, RG mainly function as active NSCs that continuously proliferate and generate newborn neurons under both physiological and regenerative conditions (März et al., 2010; Kroehne et al., 2011; Rothenaigner et al., 2011). The molecular mechanisms that regulate adult and regenerative neurogenesis by RG in the telencephalon have been investigated comprehensively using pharmacological inhibitors, transgenic strains, and transcriptome analyses (Kizil et al., 2012; Cosacak et al., 2019; Demirci et al., 2020; Lange et al., 2020). On the other hand, most of RG are quiescent, while neuroepithelial-like stem cells contribute to adult neurogenesis in the adult optic tectum (Ito et al., 2010). Previous studies have shown that stab wound injury in the optic tectum induces RG proliferation and differentiation into neurons in young adult zebrafish (2–4 months old) under regenerative conditions (Shimizu et al., 2018; Ueda et al., 2018; Yu and He, 2019), but induces RG proliferation without neurogenesis in elderly adult zebrafish (6–12 months old) (Lindsey et al., 2019). Moreover, Yu and He found that only a few neurons were generated from RG using long-term EdU labeling and Cre-loxP-based clonal analysis. The analysis of gene expression changes in RG using single-cell RNA-sequencing (RNA-seq) at 3 days post-injury (dpi) showed that Notch signaling inhibition promotes RG proliferation and differentiation. However, few studies have investigated gene expression changes that trigger RG proliferation during the early stages of tectum regeneration compared to the retina and telencephalon (Sifuentes et al., 2016; Demirci et al., 2020).

During the early stages after CNS damage, inflammatory responses commonly occur in both mammals and teleosts (Kizil et al., 2015; Buscemi et al., 2019; Lambertsen et al., 2019). In the mouse CNS, both the negative and positive effects of inflammatory responses on tissue regeneration, have been well studied in order to improve tissue damage and neurodegenerative disorders (Shichita et al., 2014; Rajkovic et al., 2018; Otani and Shichita, 2020). In zebrafish, inflammatory responses are required during brain regeneration to induce RG cell proliferation and differentiation (Kyritsis et al., 2012; Ueda et al., 2018), and the acute inflammation induced by zymosan A injection is sufficient to induce RG proliferation without injury (Kyritsis et al., 2012). Although multiple studies have analyzed the function of inflammatory mediators, such as cytokines and leukotriene, in the zebrafish telencephalon and retina (Kyritsis et al., 2012; Zhao et al., 2014; Bhattarai et al., 2016), the molecular mechanisms related to inflammatory signaling that trigger RG proliferation following optic tectum injury remain poorly understood.

In this study, we analyzed the cellular and transcriptomic changes and molecular mechanisms regulating RG proliferation during the early stages of tectum regeneration. Together, the findings of these transcriptomic and functional analyses revealed that IL6/Stat3 signaling is an initial trigger of RG activation during optic tectum regeneration. Therefore, components of molecular mechanisms that cooperate with IL6/Stat3 signaling during tissue regeneration could be promising molecular targets to enhance the regenerative capacity of the human CNS.

## Materials and Methods

### Zebrafish Strains

Zebrafish (*Danio rerio*) were maintained according to standard procedures (Westerfield, 2007). All experiments were performed according to protocols approved by the Institutional Animal Care and Use Committee of the National Institute of Advanced Industrial Science and Technology and Waseda University. The RIKEN Wako (RW) wild-type strain was obtained from the Zebrafish National BioResource Center of Japan^1^. The *Tg(gfap:GFP)^*mi*2001^* strain with RG-specific GFP expression (Bernardos and Raymond, 2006) was obtained from the Zebrafish International Resource Center (ZIRC). Three-to-four-month-old RW and *Tg*(*gfap:GFP*) fish were used for all experiments.

### Stab Wound Injury

Zebrafish were anesthetized using 0.02% tricaine (pH 7.0; Nacalai Tesque, Kyoto, Japan) and a 30-gauge (30G) needle (Terumo, Tokyo, Japan) was inserted vertically into the right hemisphere of the optic tectum through the boundary of two bones on the right optic tectum, as described previously (Shimizu et al., 2018). The uninjured left hemisphere was considered an internal control. The 30G needle was inserted to a depth of approximately 0.75 mm, almost equal to half the length of the 30G bevel. For gene expression analyses, such as RNA-seq and quantitative real-time PCR, both hemispheres of the optic tectum were injured and fixed at each time point.

### Histology and Immunohistochemistry

For immunohistochemistry (IHC), zebrafish were anesthetized using 0.02% tricaine and perfused intracardially with Ringer’s solution, followed by 4% paraformaldehyde (PFA; FUJIFILM Wako, Osaka, Japan) solution. After the brains had been dissected, they were fixed in 4% PFA overnight at 4°C, transferred into 20% sucrose overnight at 4°C for cryopreservation, and then embedded in a 2:1 mixture of 20% sucrose and O.C.T. compound (Tissue-Tek, Torrance, CA, United States). The brain tissues were then cut into 14 μm thick sections using a cryostat (CM1850, Leica, Wetzlar, Germany) and IHC was performed as described previously (Shimizu et al., 2018) with the following For primary antibodies: mouse anti-proliferating cell nuclear antigen (PCNA, 1:200, sc-56, Santa Cruz Biotechnology, Dallas, TX, United States), rabbit anti-brain lipid binding protein (BLBP, 1:500, ABN14, Millipore, Burlington, MA, United States), and Dylight 594-conjugated isolectin B4 (IB4, 1:200, Vector Laboratories, Burlingame, CA, United States). Alexa Fluor 488- and 568-conjugated subclass-specific secondary antibodies (1:500, Invitrogen, Carlsbad, CA, United States) were used. For PCNA immunodetection, antigen retrieval was performed by incubating slides in 10 mM sodium citrate for 30 min at 85°C before treatment with the primary antibody. For nuclear staining, the samples were incubated in Hoechst 33258 (1:500, FUJIFILM Wako) for 30 min after IHC. Sections were embedded in PermaFluor (Thermo Fisher Scientific, Waltham, MA, United States).

### RNA Sequencing

Zebrafish with or without stab wound injuries were anesthetized using 0.02% tricaine and both hemispheres of the optic tectum were dissected. For RNA-seq, total RNA was extracted from three pooled individuals using TRIzol reagent (Invitrogen) and purified using a Directo-zol RNA Miniprep kit (Zymo Research, Irvine, CA, United States) for each biological replicate. All RNA samples were sent to Macrogen Japan (Tokyo, Japan) and libraries were prepared from samples with RIN > 7.0 using NovaSeq6000. RNA-seq was performed in triplicate for each group using NovaSeq6000 (Illumina, CA, United States) with 2 × 100 bp paired ends. Sequencing data from the intact optic tectum was used as a control for the injured tectum at 6, 12, and 24 h post-injury (hpi).

### Bioinformatics Analysis

The quality of the raw data produced from each sample was checked using FastQC (v0.11.5) and low-quality reads (<Q20) and adapter sequences were removed using Trimmomatic-0.33 (Bolger et al., 2014). Trimmed and filtered reads were aligned to the zebrafish reference genome (Ensembl, GRCz11) using HISAT2 (v2.0.5) (Kim et al., 2015) before being assembled and counted using FeatureCounts (v1.6.3) (Liao et al., 2014). The iDEP.82 pipeline (Ge et al., 2018) was used to normalize read counts and DESeq2 (Love et al., 2014) was used to identify differentially expressed genes (DEGs) with fold change >2 in both directions and a Benjamini-Hochberg adjusted *p*-value (FDR) < 0.05 between intact and injured tectum at 6, 12, and 24 hpi. The Matascape pipeline (Zhou et al., 2019) (min overlap: 3, *p*-value cutoff: 0.01, min enrichment: 1.5) was used to perform enrichment analyses based on the DEGs using Gene Ontology (GO) terms, Kyoto Encyclopedia of Genes and Genomes (KEGG) pathways (Kanehisa and Goto, 2000), and Reactome pathways (Fabregat et al., 2018).

### Quantitative Real-Time PCR

Zebrafish with or without stab wound injuries were anesthetized using 0.02% tricaine and both hemispheres of the optic tectum were dissected. Total RNA from each fish was purified using a Directo-zol RNA Miniprep kit and cDNA was synthesized using ReverTra Ace with gDNA remover (Toyobo, Osaka, Japan). Quantitative real-time PCR (qRT-PCR) was performed using gene-specific primers for *actb2*, *gfap*, *il6*, *il11a*, *il11b*, *jak1*, and *stat3*, as listed in Supplementary Table 5.

### Flow Cytometory and Cell Sorting

Zebrafish were anesthetized using 0.02% tricaine and both hemispheres of the injured optic tectum were dissected. The samples dissected from three zebrafish were pooled in HBSS (FUJIFILM Wako) which was subsequently replaced with PBS containing 0.4% glucose, 0.04% BSA (Sigma-Aldrich, St. Louis, MO, United States), and 0.04% L-cysteine with 0.5% DNase and 0.5% papain. After incubation at 37°C for 5 min, the pooled sample was washed three times with DMEM (Sigma-Aldrich) containing 10% horse serum (Vector Laboratories), 20 μM L-glutamine, and 10 μM sodium pyruvate. After dissociation by pipetting and centrifugation at 300 × *g*, the pellets were resuspended in PBS and filtered through a 25 μm cell strainer. Cell sorting was performed using a FACSAria^^TM^ III (BD): 450,000 of the GFP^high^ population and GFP^neg^ population in *Tg(gfap:GFP)* were sorted separately into PBS with 3% FBS. For qRT-PCR, RNA was extracted from sorted cells and synthesized into cDNA using CellAmpTM Direct Lysis and RT kits (Takara Bio, Shiga, Japan). To obtain sufficient RNA for cDNA synthesis, extracted RNA was concentrated by precipitation with a Gene-Packman Coprecipitant (Nacalai Tesque).

### Drug Administration and Cerebroventricular Microinjection

To inhibit Stat3 signaling, injured zebrafish were maintained in water containing 10 or 100 μM S3I-201 (Selleck, Houston, TX, United States) (Liang et al., 2012) dissolved in 1% dimethyl sulfoxide (DMSO, FUJIFILM Wako) for 24 h. To activate Stat3 signaling without injuring the tectum, 100 ng/μL human IL6 (PeproTech, Cranbury, NJ, United States) was injected into the cerebrospinal fluid as described previously (Kizil and Brand, 2011).

### Cell Quantification

To quantify proliferative RG in the adult optic tectum, we counted the number of BLBP+ PCNA+ cells imaged using a confocal microscope C1 plus (Nikon) with UPlanSApo 20 × (NA 0.70) and 40 × (NA 0.95) objectives. To quantify proliferated RG after stab injury, we counted the number of BLBP+/PCNA+ cells in 3–5 serial sections (every other section) around the center of the injury and also in the uninjured hemisphere as an internal control. To quantify RG proliferation in the intact tectum or tectum treated with PBS or IL6 microinjection, we counted the number of BLBP+ PCNA+ cells in 5–10 serial sections (every other section) in a similar area to the injured tectum.

### Statistical Analysis

All experimental data were expressed as the mean ± standard error of the mean (SEM). Sample numbers are provided in the figure legends. Statistical analysis between two groups was performed using paired or unpaired Student’s *t*-tests. When comparing three or more groups, one-way analysis of variance (ANOVA) was performed followed by Tukey’s *post hoc* test. *P*-values were calculated using GraphPad Prism and were presented as follows: ^∗∗∗^*p* < 0.001; ^∗∗^*p* < 0.01; and ^∗^*p* < 0.05.

## Results

### Stab Wound Injury Induced Macrophage Migration and RG Proliferation in the Adult Optic Tectum Within 24 h

**FIGURE 1:**
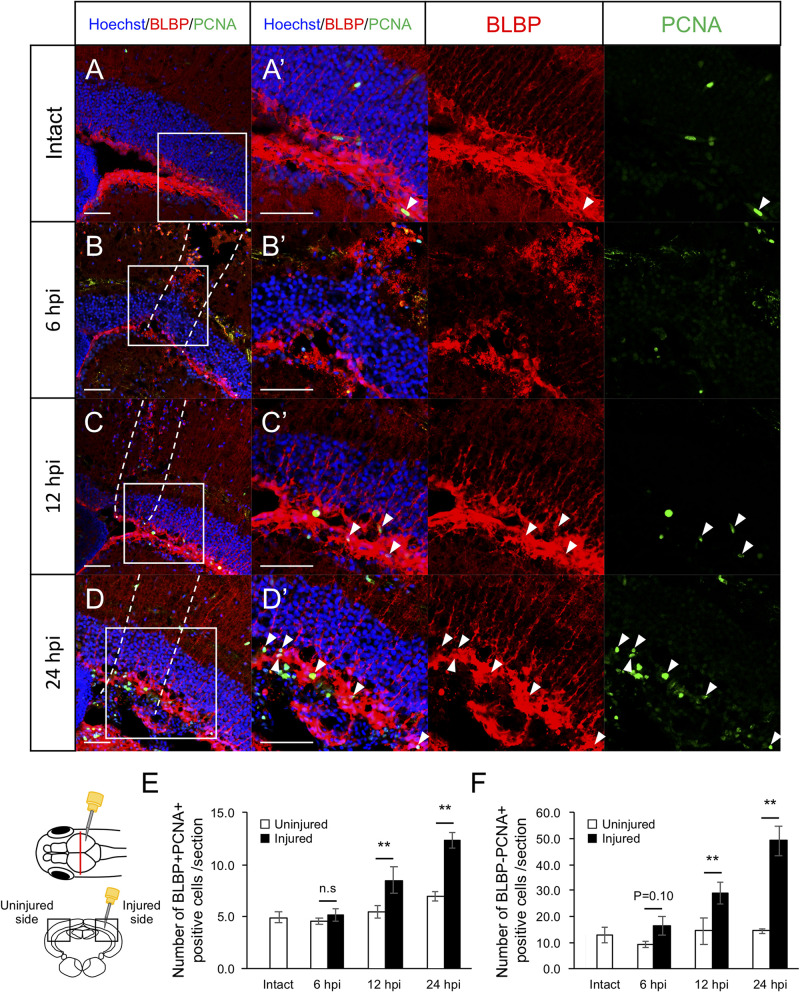
Induction of RG proliferation during the early stages after stab wound injury. **(A–D)** Representative images of proliferative RG (BLBP+ PCNA+) in intact fish **(A)** and injured fish during the early stages of regeneration, 6, 12, and 24 hpi **(B–D)**. **(A’–D’)** Magnified images of the boxed area in each image. White arrowheads indicate BLBP+ PCNA+ cells and dashed lines **(B–D)** indicate area injured by needle insertion. Scale bar: 100 μm in **(A–D)**, 50 μm in **(A’–D’)**. **(E)** Quantification of proliferative RG in intact fish (*n* = 4) and on the uninjured and injured hemispheres of injured fish at 6, 12, and 24 hpi. Statistical analyses between uninjured and injured hemispheres at each time point were evaluated using paired Student’s *t*-tests. **(F)** Quantification of proliferative cells except RG (BLBP-PCNA+) in intact fish (*n* = 4) and the uninjured and injured hemispheres of injured fish at 6, 12, and 24 hpi. Statistical analyses between uninjured and injured hemispheres at each time point were evaluated using paired Student’s *t*-tests.

**FIGURE 2:**
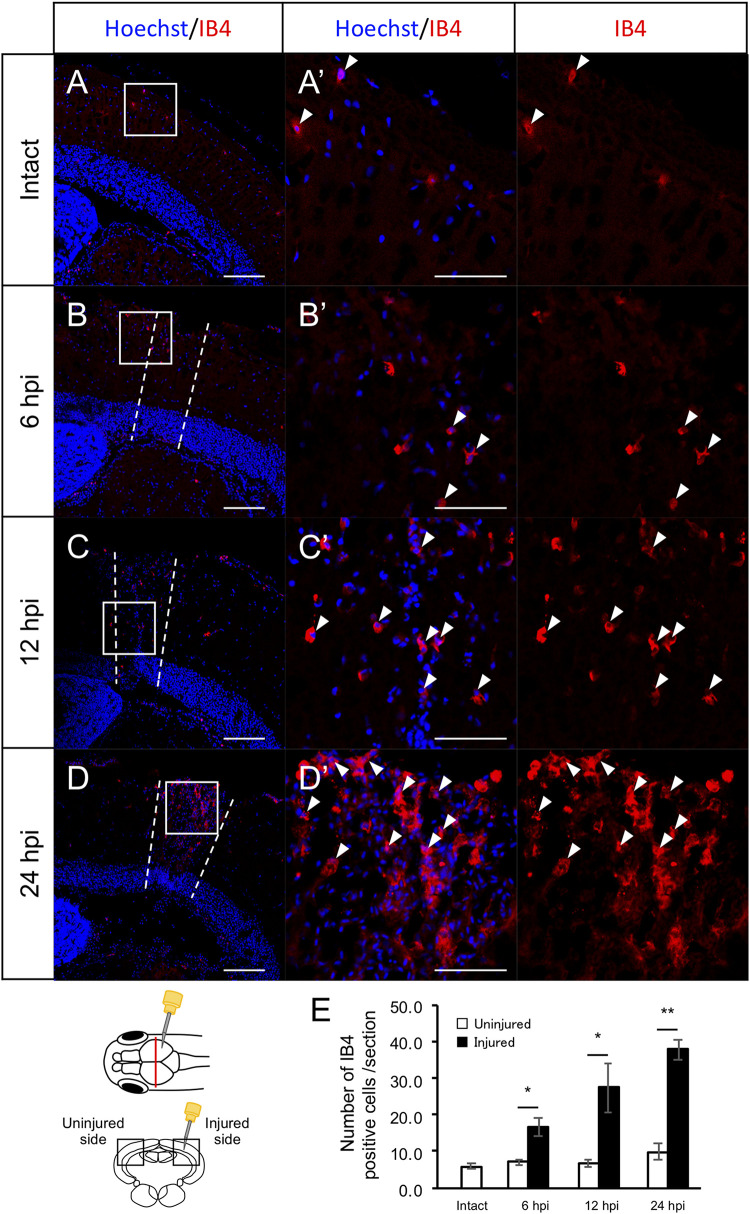
Induction of IB4+ macrophage migration during the early stages after stab wound injury. **(A–D)** Representative images of IB4-positive macrophages in intact fish **(A)** and the injured hemisphere of injured fish during the early stages of regeneration, 6, 12, and 24 hpi **(B–D)**. **(A’–D’)** Magnified images of the boxed area in each image. White arrowheads indicate BLBP+ PCNA+ cells and dashed lines **(B–D)** indicate the area injured by needle insertion. Scale bar: 100 μm in **(A–D)**, 50 μm in **(A’–D’)**. **(E)** Quantification of IB4-positive cells in intact fish (*n* = 4) and the uninjured and injured hemispheres of injured fish at 6, 12, and 24 hpi. Statistical analyses between uninjured and injured hemispheres at each time point were evaluated using paired Student’s *t*-tests.

Previously, we found that RG proliferation was induced by stab wound injury in the adult optic tectum (Shimizu et al., 2018) while other studies have shown that immune responses during the early stages after stab wound injury are required for RG proliferation in the adult telencephalon and optic tectum (Kyritsis et al., 2012; Ueda et al., 2018). Although molecular pathways that work in RG after the tectum injury have been well investigated by pharmacological experiments, transgenic lines and single cell RNA-seq (Shimizu et al., 2018; Ueda et al., 2018; Lindsey et al., 2019; Yu and He, 2019; Kiyooka et al., 2020), the global changes in gene expression related to immune activation and RG proliferation during the early stages of optic tectum regeneration had not yet been investigated. In this study, we first determined the time points for RNA-seq by evaluating RG and macrophage responses at 6, 12, and 24 hpi. After immunostaining for BLBP, an RG marker, and PCNA, a proliferative cell marker at 6, 12, and 24 hpi (Figures 1A–D and Supplementary Figure 1), we quantified BLBP+ PCNA+ cells in intact (control) and injured optic tectum (injured hemisphere and contralateral uninjured hemisphere; Figure 1E). At 6 hpi, the number of proliferative RG cells in the two hemispheres of the optic tectum did not significantly differ (Figure 1E); however, significant RG proliferation was observed on the injured side at 12 hpi in comparison to the contralateral uninjured hemisphere. Furthermore, the number of BLBP-PCNA+ cells, which likely include immune cells such as microglia and macrophages (Lindsey et al., 2019; Yu and He, 2019), also significantly increased from 12 to 24 hpi (Figure 1F). To examine macrophage responses after injury, we performed immunostaining using an IB4 antibody conjugated with Dylight 594 (Lai et al., 2017) in the intact and injured optic tectum at 6, 12, and 24 hpi (Figures 2A–D and Supplementary Figure 2). Few IB4-positive cells were observed in the intact optic tectum (Figures 2A,E), suggesting that IB4-positive cells were not microglia as there were fewer of these cells in the intact optic tectum than GFP-positive cells in *Tg*(*mpeg1:GFP*) (Lindsey et al., 2019; Yu and He, 2019). Interestingly, we found that there were significantly more IB4-positive cells in the injured hemisphere than in the uninjured hemisphere at 6, 12, and 24 hpi. Together, these results indicate that during the early stages of tectum regeneration, innate immune activation such as macrophage migration is induced at 6 hpi, followed by the activation of RG proliferation at 12 and 24 hpi.

### Comprehensive Transcriptomic Analyses During the Early Stages of Optic Tectum Regeneration

**FIGURE 3:**
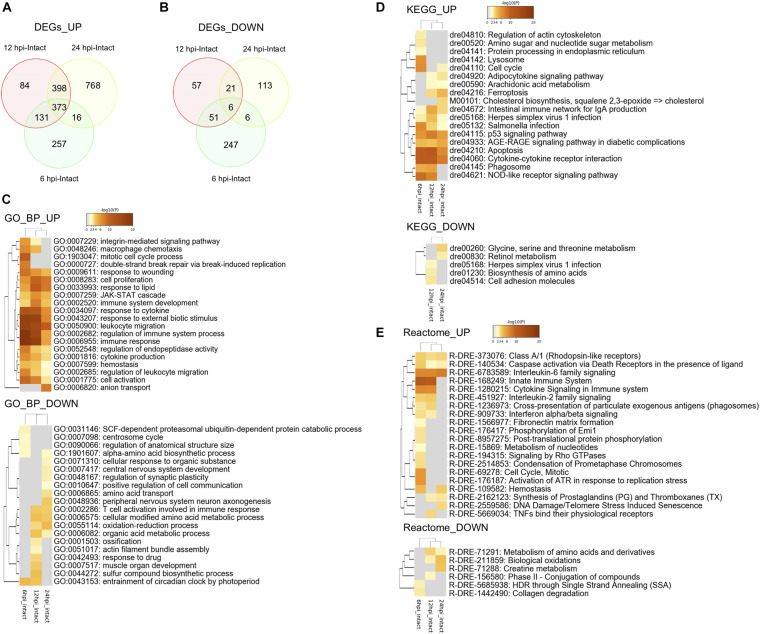
Transcriptomic and enrichment analyses during the early stages of optic tectum regeneration. **(A,B)** Venn diagram indicating upregulated **(A)** and downregulated **(B)** differentially expressed genes (DEGs) identified by DEseq2 between injured (6, 12, and 24 hpi) and intact optic tectum. Fold change >2 in both directions and FDR < 0.05. **(C–E)** Heatmaps indicating clusters of significantly enriched GO biological process (BP) **(C)**, KEGG pathway **(D)**, and Reactome pathway **(E)** terms based on DEGs between the injured and intact tectum (6, 12, and 24 hpi) using Metascape enrichment analysis. UP and DOWN indicate enrichment analyses based on up- and downregulated DEGs, respectively.

Since macrophage migration into the injured tectum was observed at 6 hpi and RG proliferation was induced at 12 hpi (Figures 1E, 2E), we investigated changes in gene expression related to macrophage migration and the induction of RG proliferation during the early stages of tectum regeneration. Comprehensive transcriptome analysis was performed between the intact and injured optic tectum at 6, 12, and 24 hpi and DEGs were quantified at each time point (fold change >2 in both directions and FDR < 0.05) to identify the major responses during early tectum regeneration. We identified a total of 1,087 DEGs [777 upregulated (UP), 310 downregulated (DOWN)] at 6 hpi, 1,121 DEGs (986 UP, 135 DOWN) at 12 hpi, and 1,711 DEGs (1,565 UP, 146 DOWN) at 24 hpi (Figures 3A,B). The top 100 up- or downregulated DEGs at each time point are shown in Supplementary Table 1. Next, we analyzed pathways enriched for up- or downregulated DEGs at 6, 12, and 24 hpi using Metascape enrichment analysis for GO terms, KEGG pathways, and Reactome pathways (Supplementary Figures 3–5 and Supplementary Tables 2–4). The enriched GO terms for biological processes (BP) based on the upregulated DEGs at 6, 12, and 24 hpi (Figure 3C, GO_UP) included “response to wounding,” “cell proliferation,” “JAK-STAT cascade,” “hemostasis” and some GO terms related to cytokines and the immune system. In addition, KEGG pathway analyses based on the upregulated DEGs (Figure 3D, KEGG_UP) included “cell cycle,” “p53 signaling pathway,” “AGE-RAGE signaling pathway in diabetic complication,” “apoptosis” and cytokine-related pathways such as “adipocytokine signaling pathway” and “cytokine-cytokine receptor interaction.” Reactome analyses of the upregulated DEGs (Figure 3E, Reactome_UP) also included “interleukin-6 (IL6) family signaling,” “caspase activation,” “hemostasis,” and some pathways related to the immune system and cytokine signaling. When the same analyses were performed based on downregulated DEGs, amino acid metabolism was the only enriched pathway shared between GO BP, KEGG, and Reactome analysis. Therefore, these pathway analyses based on DEGs within 24 h after optic tectum injury suggest that immune activation, cell proliferation, apoptosis, and cytokine signaling, including the IL6/Jak-Stat pathway, are major responses during the early stages of tectum regeneration.

### IL6/Jak1-Stat3 Signaling Is Activated During the Early Stages of Optic Tectum Regeneration

**FIGURE 4:**
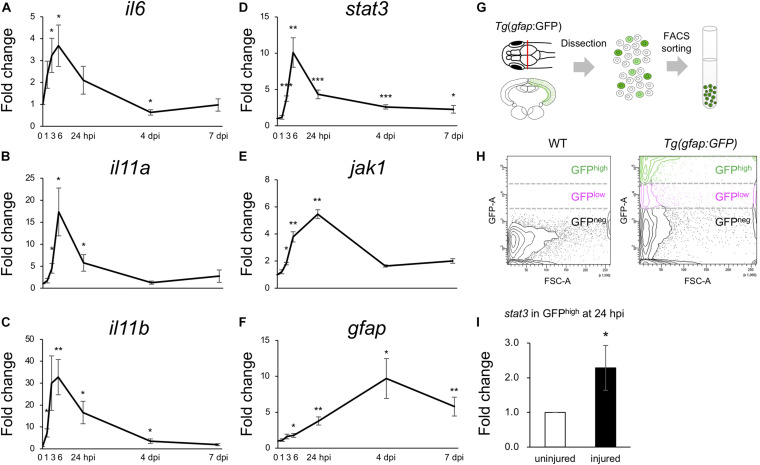
Activation of IL6/Stat3 signaling during the early stages of optic tectum regeneration. **(A–F)** Quantitative real-time (qRT-) PCR analyses of IL6 superfamily cytokines *il6* **(A)**, *il11a* **(B)**, and *il11b* **(C)**, and Jak/Stat signaling components *stat3* **(D)**, *jak1* **(E)**, and *gfap* **(F)**. Each graph indicates relative gene expression in the injured tectum from 1 to 7 dpi compared to intact optic tectum (*n* = 4 per gene per time point). Statistical analyses between intact and injured hemispheres at each time point were evaluated using unpaired Student’s *t*-tests. **(G)** Schematic diagram of the cell sorting workflow using the RG-specific reporter line, *Tg(gfap:GFP)*. **(H)** Representative images of FACS plots for GFP-positive cells from the optic tectum of wild-type (left) or *Tg(gfap:GFP)* (right) fish. **(I)** Quantification of *stat3* expression in GFP^high^-positive cells from uninjured and injured optic tectum at 24 hpi using qPCR. The graph shows relative *stat3* expression in GFP^high^-positive cells from injured tectum at 24 hpi compared to uninjured tectum (*n* = 11). Statistical analyses between uninjured and injured hemispheres was evaluated using unpaired Student’s *t*-tests.

Previous studies have shown that *stat3* is upregulated in Müller glia during the early stages (8 and 16 hpi) of retinal regeneration (Sifuentes et al., 2016) and that injection with *stat3* morpholino antisense oligonucleotides can suppress the proliferation of Müller glia after retinal injury (Nelson et al., 2012). However, the functions of IL6/Jak1-Stat3 signaling during optic tectum regeneration have not yet been studied. Since the upregulated DEGs during the early stages of tectum regeneration were enriched for IL6 family cytokines and Jak-Stat signaling (Figures 3C,E), we quantified changes in the expression of *il6*, *il11a*, and *il11b* (IL6 family cytokines in the top 100 upregulated DEGs at 6, 12, or 24 hpi) and *stat3*, *jak1*, and *gfap* (Jak-Stat signaling) at different time points from 1 to 7 dpi (Figures 4A–F). Changes in *il6*, *il11a*, *il11b*, and *stat3* expression peaked around 6 hpi, after which *jak1* and *gfap* were upregulated. To further examine the changes in *stat3* expression in RG during the early stages of optic tectum injury, we isolated RG from *Tg*(*gfap:GFP*), which display RG-specific GFP expression (Figures 4G,H and Supplementary Figure 6). Notably, *stat3* expression was significantly higher in GFP^high^-positive cells than in the intact reporter fish at 24 hpi (Figure 4I). Moreover, we confirmed that the IL6 receptors *il6r* and *il6st* were expressed in GFP^high–^positive cells using RT-PCR (data not shown). Taken together, these results suggest that the upregulation of IL6 family cytokines stimulates Stat3 signaling in RG during the early stages of optic tectum regeneration.

### Stat3 Inhibitors Suppress RG Proliferation After Stab Wound Injury

**FIGURE 5:**
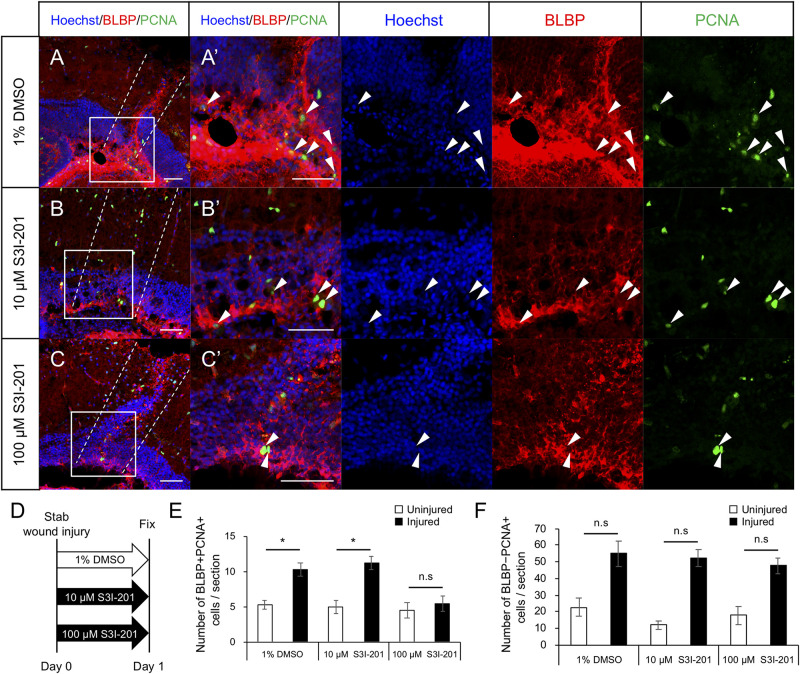
Stat3 inhibitor S3I-201 suppresses RG proliferation after stab wound injury in adult optic tectum. **(A–C)** Representative images of proliferative RG (BLBP+ PCNA+) in injured optic tectum treated with 1% DMSO **(A)**, 10 μM S3I-201 **(B)**, or 100 μM S3I-201 **(C)** at 1 dpi. **(A’–C’)** Magnified images of the boxed area in each image. White arrowheads indicate BLBP+ PCNA+ cells and dashed lines indicate the area injured by needle insertion. Scale bar: 50 μm in **(A–C)** and **(A’–C’)**. **(D)** Schematic diagram of drug administration. **(E)** Quantification of proliferative RG (BLBP+ PCNA+) treated with 1% DMSO, 10 μM S3I-201 (*n* = 4), or 100 μM S3I-201 (*n* = 5) at 1 dpi. Statistical analyses were performed using one-way ANOVA with Tukey’s *post hoc* test. **(F)** Quantification of proliferative cells except RG (BLBP-PCNA+) treated with 1% DMSO, 10 μM S3I-201 (*n* = 4), or 100 μM S3I-201 (*n* = 5) at 1 dpi. Statistical analyses were performed using one-way ANOVA with Tukey’s *post hoc* test.

To examine whether Stat3 signaling activation is required for RG proliferation after tectum injury, we maintained injured zebrafish in water containing the Stat3 inhibitor S3I-201 (10 or 100 μM) or 1% DMSO for 1 day and performed immunostaining for BLBP and PCNA at 24 hpi to quantify cell proliferation (Figures 5A–D). The number of proliferative RG (BLBP+ PCNA+)was significantly lower in fish treated with 100 μM S3I-201 than in those treated with 1% DMSO or 10 μM S3I-201 (Figure 5E), suggesting that S3I-201 inhibited RG proliferation after tectum injury in a dose-dependent manner. However, the number of BLBP-PCNA+ cells, likely including immune cells, was not significantly changed by S3I-201 treatment (Figure 5F). Moreover, we confirmed that Stat3 inhibitor does not significantly affect NE proliferation in the intact zebrafish (Supplementary Figure 7). Taken together, these results suggest that Stat3 inhibitor specifically suppressed RG proliferation and that Stat3 signaling is required to induce RG proliferation after stab wound injury in the optic tectum.

### Cerebroventricular Microinjection of IL6 Recombinant Protein Induces RG Proliferation Without Stab Wound Injury

**FIGURE 6:**
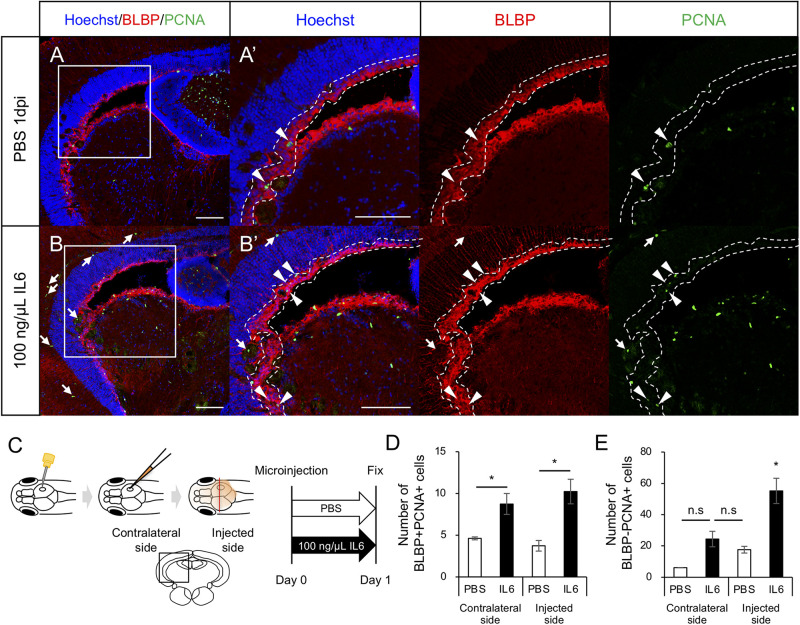
IL6 induces RG proliferation without stab wound injury in adult optic tectum. **(A,B)** Representative images of proliferative RG (BLBP+ PCNA+) in the contralateral side with PBS or 100 ng/μL IL6 at 1 dpi. Allow heads indicate BLBP+ PCNA+ cells and arrows indicate BLBP- PCNA+ cells. Scale bar: 100 μm in **(A–D)** and **(A’,B’)**. **(C)** Schematic diagram of cerebroventricular microinjection. A small hole was made in the center of the skull above the right hemisphere of the optic tectum using a 30G needle. The injected solution spread through the corticospinal fluid. **(D)** Quantification of proliferative RG (BLBP+ PCNA+) in the injected and contralateral hemisphere with PBS (*n* = 4) or 100 ng/μL IL6 (*n* = 3) at 1 dpi. **(E)** Quantification of BLBP- PCNA+ cells in the injected and contralateral hemisphere with PBS or IL6 at 1 dpi. Statistical analyses were performed using one-way ANOVA with Tukey’s *post hoc* test.

To determine whether IL6 was sufficient to induce RG proliferation under physiological conditions, we injected human IL6 recombinant protein (100 ng/μL) or PBS into the cerebrospinal fluid (Figure 6C) and performed immunostaining on fixed samples using anti-BLBP and anti-PCNA antibodies to quantify proliferating RG. We respectively counted the number of BLBP+ PCNA+ cells in the injected and contralateral hemispheres and found that the IL6 injection significantly increased the number of proliferating RG in the contralateral hemisphere compared to that with PBS injection (Figures 6A,B,D). We also counted the number of BLBP-PCNA+ cells and confirmed that the number of BLBP− PCNA+ cells except NE was not significantly increased in the contralateral hemisphere (Figure 6E) and that IL6 injection had no significant effect in the NE proliferation (data not shown). In the injected hemisphere, we found the small incision by glass capillary compared to that by the 30G needle (data not shown). The stab wound injury by the 30G needle did not significantly increase both BLBP+ PCNA+ and BLBP-PCNA+ cells in the contralateral hemisphere (Figures 1E,F), suggesting that the smaller incision in the injected hemisphere also have no significant effects on cell proliferation in the contralateral side. Taken together, these results suggest that IL6 is sufficient to induce RG proliferation in the adult zebrafish optic tectum without stab wound injury. Transcriptomic analyses and functional analyses reveal that IL6/Stat3 signaling is an important initial trigger of RG proliferation at the early stages of tectum regeneration.

## Discussion

Zebrafish display a superior capacity for neuronal regeneration in the CNS and the molecular mechanisms that regulate regenerative neurogenesis in the telencephalon and optic tectum have been well studied; however, few comprehensive transcriptomic analyses have been performed to study regenerating optic tectum. In this study, we attempted to elucidate the triggers that stimulate RG proliferation in response to optic tectum injury, with a particular focus on the early stages of tectum regeneration. First, we confirmed that the levels of IB4+ macrophages increased at 6 hpi, followed by the induction of RG proliferation, while transcriptomic analyses based on DEGs revealed that apoptosis, the immune system, cytokine signaling, cell proliferation, and IL6/Jak-Stat signaling were upregulated during the early stages of tectum regeneration. After validating that *il6* and *stat3* expression peaked at 6 hpi and that *jak1* expression peaked at 24 hpi, we revealed that Stat3 inhibition suppressed RG proliferation at 1 dpi and that microinjecting human recombinant IL6 into cerebroventricular fluid induced RG proliferation without injury. Together, our findings demonstrate that IL6/Stat3 signaling is an initial trigger of RG activation during optic tectum regeneration and that transcriptome analyses are powerful tools for investigating molecular mechanisms.

### Macrophage Migration Prior to RG Proliferation After Optic Tectum Injury

Previous studies have shown that the activation of innate immune responses is required for tissue regeneration in both the CNS and other tissues, such as the heart and fin (Kyritsis et al., 2012; Petrie et al., 2014; Lai et al., 2017). In the optic tectum, dexamethasone-induced immunosuppression also suppresses RG cell proliferation after stab wound injury (Ueda et al., 2018). Here, we labeled macrophages using IB4 antibody (Lai et al., 2017), although *Tg*(*mpeg1:GFP*)*^gl22^* (Ellett et al., 2011) and antibodies such as L-plastin and 4C4 have also been utilized to examine microglia and macrophage responses (Baumgart et al., 2012; Kyritsis et al., 2012; Oosterhof et al., 2017). Previously, Lai et al. found that IB4 staining colocalized with GFP-positive cells in *Tg(mpeg1:EGFP)* but not with GFP-positive cells in the neutrophil reporter fish, *TgBAC(mpx:GFP)^*i*114^* (Renshaw et al., 2006), while Zou et al. (2013) showed that IB4 staining colocalized with GFP-positive cells in other macrophage reporter line, *Tg(cora1a:eGFP)* (Li et al., 2012) and not with endothelial cell reporter line, *Tg(flk:GFP)* (Choi et al., 2007). Together, these findings suggest that IB4 labels macrophages but not neutrophils and vascular endothelial cells. In this study, we noted an increase in the number of IB4-positive cells from 6 h after tectum injury, followed by RG proliferation. This is consistent with the increase in 4C4 positive cells and mpeg1:GFP+ cells followed by RG proliferation previously reported in the injured telencephalon (Baumgart et al., 2012; Kanagaraj et al., 2020). These things suggest that macrophage/microglia activation triggers RG reprogramming during brain regeneration though each role of infiltrating macrophage and resident microglia still remains little known. M1 and M2 macrophage polarization and their functions have been well studied in mammals and macrophage polarization is considered a promising therapeutic target for chronic inflammation, tissue regeneration, and cancer (Noy and Pollard, 2014; Hu et al., 2015). However, improved characterization of M1/M2 macrophages/microglia in zebrafish is necessary to fully understand their contribution toward tissue regeneration in zebrafish.

### Global Gene Expression Changes and Enriched Pathways at the Early Stages of Tectum Regeneration

Our transcriptome and pathway analyses during the early stages of optic tectum regeneration suggested the upregulation of genes related to the innate immune system, apoptosis, cell proliferation, cytokine signaling, and the IL6/Jak-Stat pathway. The zebrafish innate immune system includes macrophages, neutrophils, dendritic cells, and natural killer cells and our transcriptome analyses demonstrated the upregulation of macrophage markers (*mpeg1* and *mfap4*), neutrophil markers (*lyz*, *mpx*, and *nccrp1*), and a dendritic cell marker (*spi1b*) during early tectum regeneration, suggesting innate immune system activation. Moreover, T cell markers such as *cd4-1*, *cd8a*, and *foxp3a* were not upregulated at 24 hpi, suggesting that the adaptive immune system was not activated within 24 h after the injury. Studies have shown that regulatory T (T-reg) cells expressing *foxp3a* are required for tissue regeneration in the heart, retina, and spinal cord (Hui et al., 2017), with T-reg cell depletion in the mouse brain adversely affecting neurological recovery from stroke (Ito et al., 2019). Therefore, functional analyses of innate immune cells and T-reg cells are important for understanding the high tissue regeneration capacity of zebrafish.

We also found that many genes related to cytokine signaling were upregulated at 24 hpi, including pro-inflammatory molecules such as *il1b* and *il6*, and anti-inflammatory molecules such as *il4*, *il13*, and *il10*. Although both the IL6/Stat3 and IL4/Stat6 pathways induce NSC proliferation in the retina or telencephalon (Zhao et al., 2014; Bhattarai et al., 2016), we focused on IL6/Stat3 signaling as *il6* and *stat3* expression were upregulated earlier than *il4* and *stat6* after tectum injury. Thus, these secreted molecules are promising therapeutic targets for damaged tissue regeneration and should be investigated further in future functional and translational studies.

Previous studies have shown that the reprogramming of the transcription factor *ascl1a* and related genes, such as *lin28a*, *sox2*, *stat3*, and *her4*, play important functions in the retina (Ramachandran et al., 2010; Gorsuch et al., 2017; Elsaeidi et al., 2018). Therefore, we detected *lin28a* and *stat3* in upregulated DEGs and *her4.1* and *her4.2* in downregulated DEGs, while we found that *ascl1a* (log2FC = 0.63, FDR = 0.091) and *sox2* (log2FC = 0.75, FDR < 0.001) upregulation peaked at 12 hpi. These findings suggest that *stat3*, *ascl1a*, *lin28*a, *sox2*, and *her4* play important roles in RG activation during tectum regeneration. The regulation of adult and regenerative neurogenesis by *her* family genes and Notch signaling have been well studied in the optic tectum (Dozawa et al., 2014; Ueda et al., 2018; Yu and He, 2019; Kiyooka et al., 2020); however, further studies are required to investigate the regulation of adult NSCs by *ascl1a*, *lin28a*, and *sox2* during brain regeneration.

Compared to transcriptome analyses during the early stages of telencephalon regeneration (Demirci et al., 2020), many enriched pathways, including apoptosis, the innate immune system, cell proliferation, cytokine signaling, p53 signaling, and AGE-RAGE signaling, are shared between the telencephalon and optic tectum within 24 h after stab wound injury. In the zebrafish CNS, the roles of RG and neuroepithelial-like stem cells are thought to differ in different neurogenic regions. In particular, the neurogenic potential of RG during optic tectum regeneration has been controversial. RG-specific RNA-seq or single cell RNA-seq in different regions or conditions are promising for understanding the functions of RG in the adult zebrafish CNS and further transcriptomic data may allow comparative analysis between different brain regions or different species.

### Functions of Stat3 Signaling in the Activation of NSCs During Tissue Regeneration

Based on the enrichment analyses of upregulated DEGs during the early stages of tectum regeneration, we attempted to elucidate the function of IL6 and Stat3 in RG proliferation. We found that Stat3 inhibition after tectum injury suppressed RG proliferation, while injecting recombinant human IL6 induced RG proliferation without stab wound injury, consistent with previous analyses of IL6/Stat3 signaling in the zebrafish retina (Nelson et al., 2012; Zhao et al., 2014). We confirmed the *stat3* upregulation and expression of IL6 receptors in RG using FACS sorting. Moreover, microglia specific RNA-seq (Oosterhof et al., 2017; Zambusi et al., 2020) has shown that *il6* is highly expressed in the *mpeg1*+ cells including microglia and macrophages compared to the other types of cells. These findings suggest that IL6 secreted from microglia/macrophages in the injured tectum stimulates the RG proliferation through the activation of stat3 signaling. In addition, Gorsuch et al. showed that Stat3 interacts with Ascl1a, Lin28a, and Sox2 during the retinal regeneration, while Sifuentes et al. found that *stat3* expression is upregulated in Müller glia prior to *ascl1a*, *lin28a*, and *sox2* expression using Müller glia-specific RNA-seq during the early stages of retinal regeneration (8 and 16 hpi). Consistently, we confirmed *stat3* upregulation before *ascl1a* and *sox2*, suggesting that Stat3 signaling activation is an upstream trigger for the regenerative program of NSCs. Furthermore, Stat3 signaling is known to be required for the regeneration of other tissues in zebrafish, such as the heart, hair cells, and fins (Liang et al., 2012; Fang et al., 2013; Miskolci et al., 2019), suggesting that Stat3 regulates different target genes in different tissue regeneration. In mammals, Stat3 also plays important roles in neuronal regeneration from Müller glia in retina (Jorstad et al., 2020) and embryonic stem cell pluripotency (Raz et al., 1999). During the retina regeneration in mice and chickens, inhibition of Stat3 signaling promotes neuronal differentiation of Müller glia (Todd et al., 2016; Jorstad et al., 2020), while the functions of Stat3 signaling in the neuronal differentiation of NSCs during regeneration have not been well investigated in zebrafish compared to the involvement of Stat3 signaling in the proliferation of NSCs (Nelson et al., 2012; Wan et al., 2014; Zhao et al., 2014). Therefore, it is important to investigate the molecular mechanisms that cooperate with Stat3 signaling during tissue regeneration in mammals and zebrafish in order to investigate promising molecular targets to enhance the regenerative capacity of the human CNS.

## Data Availability Statement

Raw RNA-seq data are available in the NCBI Sequenced Read Archive under link: https://dataview.ncbi.nlm.nih.gov/object/PRJNA668620 with the accession numbers SRR13370844–SRR13370855.

## Ethics Statement

The animal study was reviewed and approved by the Institutional Animal Care and Use Committee of the National Institute of Advanced Industrial Science and Technology and Waseda University.

## Author Contributions

YS and TO designed the experiments. YS performed histological and molecular experiments and bioinformatics analyses. MK performed FACS based analysis. YS, MK, and TO wrote and revised the manuscript. All authors approved the submitted version manuscript.

## Conflict of Interest

The authors declare that the research was conducted in the absence of any commercial or financial relationships that could be construed as a potential conflict of interest.

## Footnotes

1 http://www.shigen.nig.ac.jp/zebra/
