# Supplementary material for: Transcriptome Analyses Reveal IL6/Stat3 Signaling Involvement in Radial Glia Proliferation After Stab Wound Injury in the Adult Zebrafish Optic Tectum
Source: Front Cell Dev Biol. 2021 Apr 30;9:668408. doi: 10.3389/fcell.2021.668408 (PMC8119998; doi:10.3389/fcell.2021.668408)
Supplement: Supplementary file 6 [file Data_Sheet_1.docx]

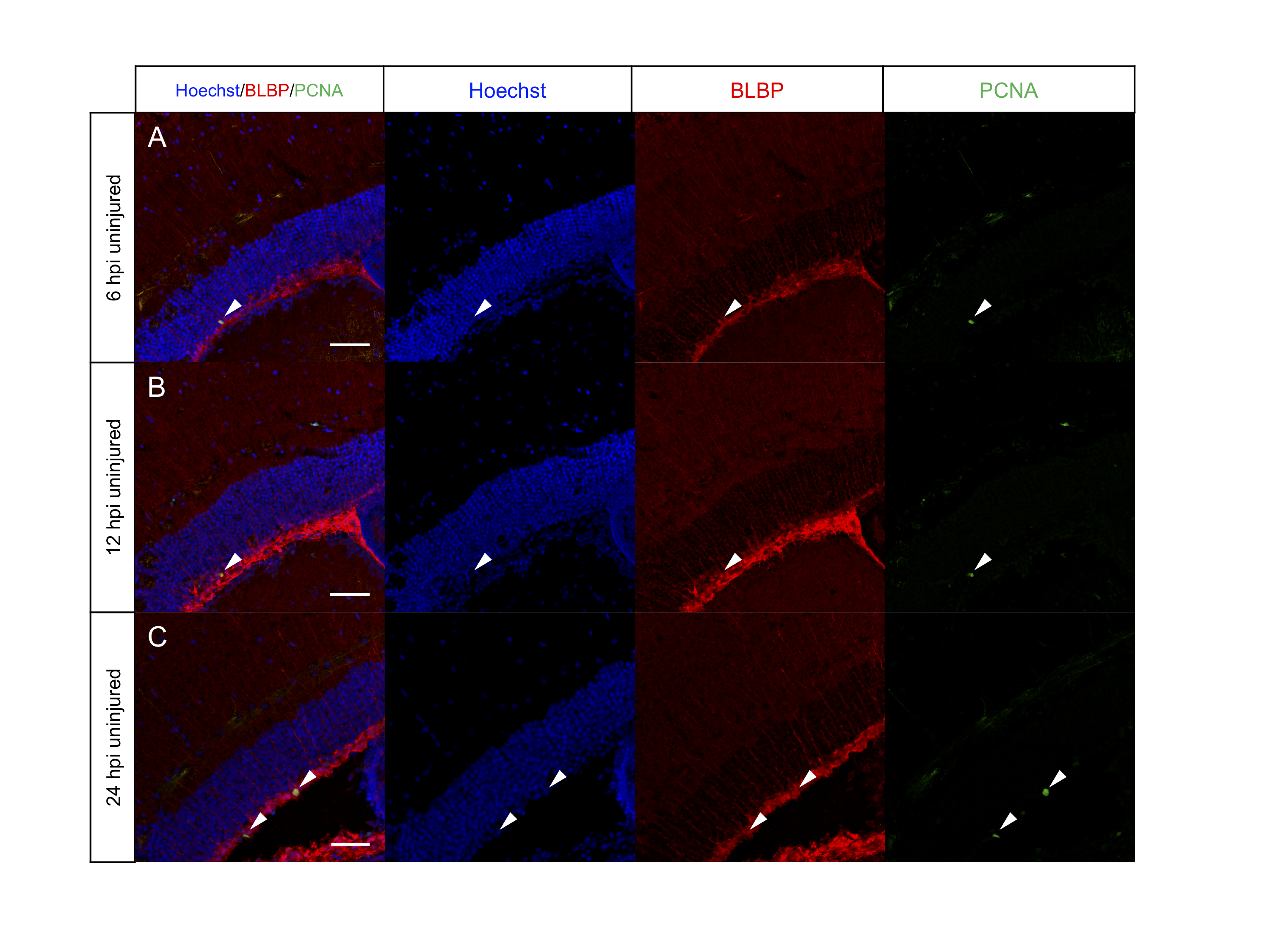
Supplementary Figures

**Supplementary Figure 1. RG proliferation in the contralateral uninjured hemisphere at 6, 12, 24 hpi**

A-C: Representative images of RG proliferation in the contralateral uninjured hemisphere at 6, 12, 24 hpi. White arrow heads indicate BLBP^+^ PCNA^+^ cells. Scale bar: 50µm in A-C


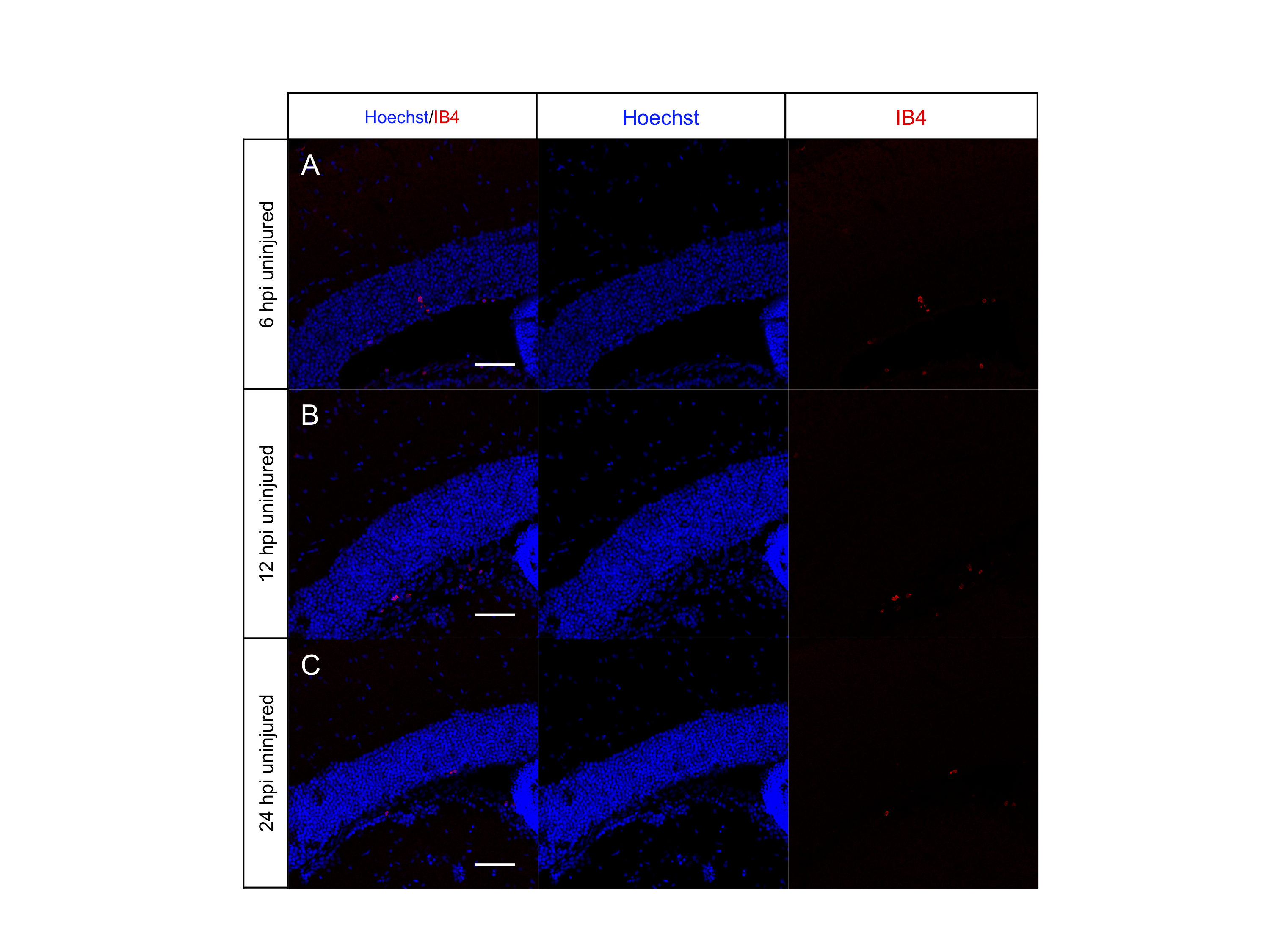


**Supplementary Figure 2. IB4 positive cells in the contralateral uninjured hemisphere at 6, 12, 24 hpi**

A-C: Representative images of IB4^+^ cells in the contralateral uninjured hemisphere at 6, 12, 24 hpi. Scale bar: 50µm in A-C

**Supplementary Figure 3. Enriched pathway analyses by using GO terms, KEEG and Reactome pathway, based on identified DEGs at 6 hpi**

Enriched pathway analyses for GO Biological process (BP), KEGG pathway and Reactome pathway with Metascape enrichment analyses (Min Overlap: 3, P Value Cutoff: 0.01, Min Enrichment: 1.5). 885 upregulated and 360 downregulated DEGs (fold change >2 in both directions and FDR <0.05) are used as the inputs. A: Enriched GO BP based on identified DEGs at 6 hpi. B: Enriched KEGG pathway based on identified DEGs at 6 hpi. No enriched pathway detected from downregulated DEGs. C: Enriched Reactome pathway based on identified DEGs at 6 hpi.

**Supplementary Figure 4. Enriched pathway analyses by using GO terms, KEEG and Reactome pathway, based on identified DEGs at 12 hpi**

Enriched pathway analyses for GO Biological process (BP), KEGG pathway and Reactome pathway with Metascape enrichment analyses (Min Overlap: 3, P Value Cutoff: 0.01, Min Enrichment: 1.5). 992 upregulated and 146 downregulated DEGs (fold change >2 in both directions and FDR <0.05) are used as the inputs. A: Enriched GO BP based on identified DEGs at 12 hpi. B: Enriched KEGG pathway based on identified DEGs at 12 hpi. C: Enriched Reactome pathway based on identified DEGs at 12 hpi.

**Supplementary Figure 5. Enriched pathway analyses by using GO terms, KEEG and Reactome pathway, based on identified DEGs at 24 hpi**

Enriched pathway analyses for GO Biological process (BP), KEGG pathway and Reactome pathway with Metascape enrichment analyses (Min Overlap: 3, P Value Cutoff: 0.01, Min Enrichment: 1.5). 1,692 upregulated and 170 downregulated DEGs (fold change >2 in both directions and FDR <0.05) are used as the inputs. A: Enriched GO BP based on identified DEGs at 24 hpi. B: Enriched KEGG pathway based on identified DEGs at 24 hpi. C: Enriched Reactome pathway based on identified DEGs at 24 hpi.

**Supplementary Figure 6. GFP positive cell sorting from radial glia reporter line, *Tg(gfap:GFP)***

A: Representative images of FACS plots of GFP positive cells from optic tectum in uninjured wild-type (left), uninjured *Tg(gfap:GFP)* (center), and injured *Tg(gfap:GFP)* at 24 hpi (right). GFP^high^ positive cells are collected for quantitative PCR. B: Ration of sorted GFP positive cells from each sample. The ration of GFP positive cell at 24 hpi was not significantly changed by stab wound injury.


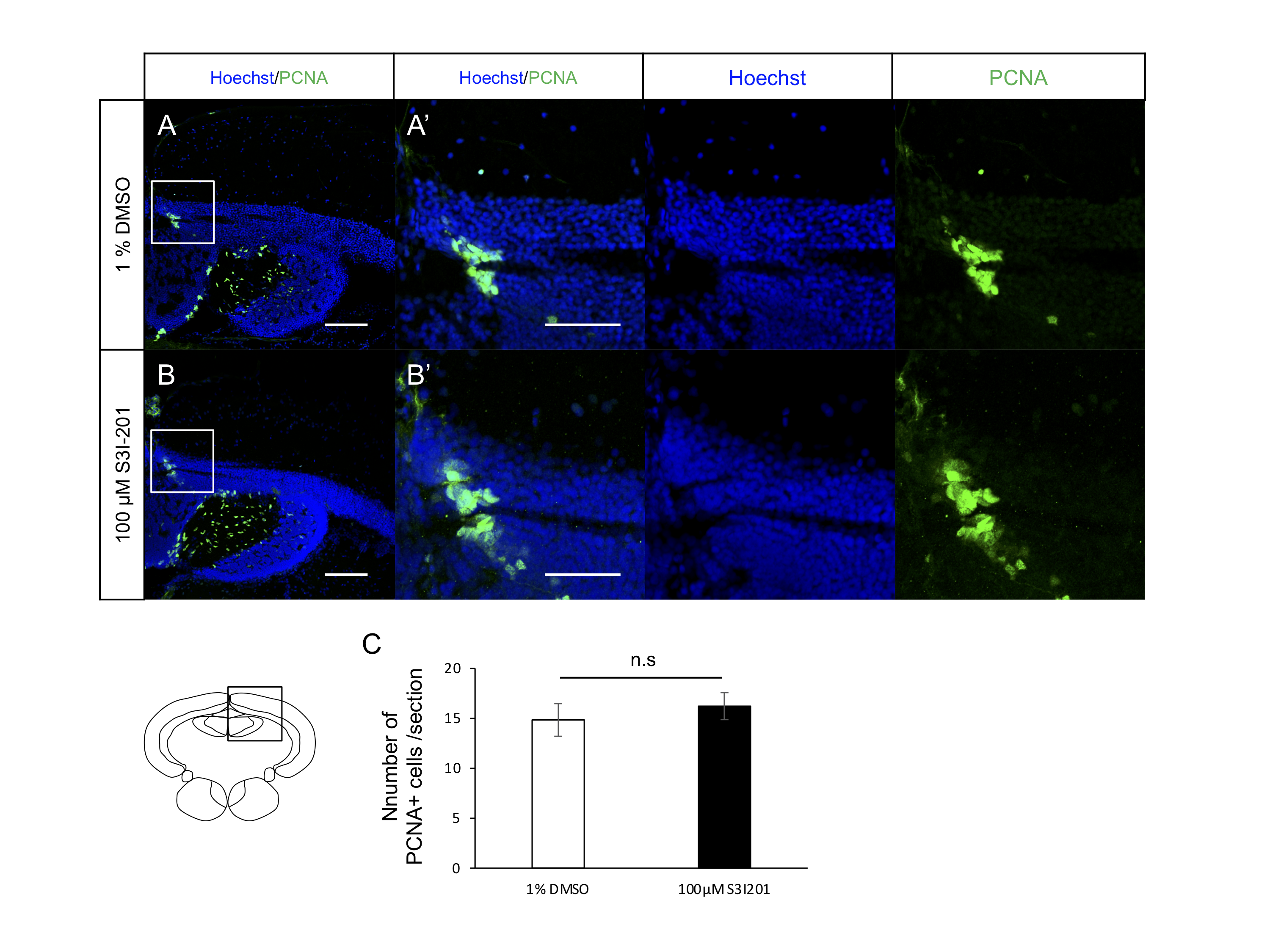
**Supplementary Figure 7. The effect of Stat3 inhibitor in the NE proliferation**

A-B: Representative images of NE proliferation with 1% DMSO or 100µM S3I-201 treatment for 1 day. Scale bar: 100µm in A-B, 50µm in A’-B’. C: Quantification of PCNA^+^ cell located in the tectal marginal zone with 1% DMSO or 100µM S3I-201 treatment for 1 day.
